# A prepartum diet supplemented with oilseeds high in oleic or linoleic acid reduced GnRH-induced LH release in dairy cows during second week postpartum

**DOI:** 10.1186/s12958-015-0060-x

**Published:** 2015-07-03

**Authors:** Reza Salehi, Marcos G. Colazo, Masahito Oba, Divakar J. Ambrose

**Affiliations:** Department of Agricultural, Food and Nutritional Science, University of Alberta, T6G 2P5 Edmonton, AB Canada; Livestock Research Branch, Alberta Agriculture and Rural Development, T6H 5 T6 Edmonton, AB Canada

**Keywords:** GnRH, LH release, Canola, Sunflower, Long chain fatty acids

## Abstract

**Background:**

The objective was to determine the effect of prepartum diets supplemented with rolled canola seed (high in oleic acid) or sunflower seed (high in linoleic acid) on luteinizing hormone (LH) pulsatility and gonadotropin releasing hormone (GnRH)-induced LH release during early postpartum.

**Methods:**

Thirty-one pregnant Holstein cows, blocked by body condition score, parity and expected calving date, were assigned to 1 of 3 prepartum diets supplemented with 8 % rolled canola or sunflower seed, or no oilseed (control) during the last 35 d of gestation. Blood samples were collected at Weeks (wk)-3, 0, +1 and +2, relative to calving, to determine non-esterified fatty acids (NEFA), Beta-hydroxy butyric acid (BHBA) and glucose. Additional blood samples were collected during wk1 (n = 5 per treatment) or wk2 (n = 5 or 6 per treatment), for 6 h, to measure LH pulsatility; thereafter, 100 mcg GnRH was administrated i.m., and blood was sampled for 4 h more, to measure GnRH-induced LH release.

**Results:**

Dietary treatment did not affect prepartum energy balance, but cows fed the control diet were in a deeper state of negative energy balance during wk2, than those fed canola (P = 0.03) or sunflower (P = 0.01). Prepartum diets did not influence the mean plasma concentration of BHBA and glucose. However, NEFA concentration during wk2 was greater in control cows than those fed sunflower (P = 0.03) or canola (P = 0.07). Prepartum diets did not affect LH pulsatility (i.e. mean, minimum, maximum concentration, pulse frequency, and amplitude during wk1 and 2). GnRH-induced LH release did not differ among dietary treatments during wk1 but the mean GnRH-induced LH release during wk2 was either greater (P = 0.02) and tended to be greater (P = 0.09) in control cows than in those fed canola and sunflower, respectively.

**Conclusions:**

Prepartum diets did not affect LH pulsatility and GnRH-induced LH release during the first week postpartum, but cows fed a diet supplemented with oilseeds high in oleic or linoleic acid released less LH than control cows, in response to an exogenous GnRH challenge during the second week postpartum.

## Background

Several studies have shown that dietary fat supplementation affects reproductive function in cattle [1]. Effects of dietary fats on fertility [2], ovarian follicular development [3] and steroidogenesis [4] have all been reported. More recently, Colazo et al. [5] reported that cows fed a prepartum diet supplemented with canola seed (high in oleic acid) had a longer interval from calving to first ovulation compared with those fed diets supplemented with either linola (high in linoleic) or flaxseed (high in linolenic). The ability of the first dominant follicle to ovulate during early postpartum is influenced by energy balance [6], postpartum health disorder [7, 8], IGF-1 concentration and LH pulsatility [9]. However, the delay in resumption of cyclicity observed in cows fed a prepartum diet supplemented with canola seed was not associated with energy balance, the incidence of health disorders or IGF-1 concentrations postpartum [5]. In addition, the diameter of the largest follicle at 7 ± 1 d after calving did not differ among dietary treatments but 25 % of cows fed a prepartum diet supplemented with canola developed ovarian follicular cysts [5] indicative of ovulatory dysfunction. It has been shown that feeding supplemental fat alters the growth dynamics of the ovarian follicle and that this effect is somewhat independent from energy [10], but whether dietary fatty acids affect the secretion of LH in ruminants is unknown. Reports indicate that fatty acid signaling may affect the neuroendocrine control of reproduction acting directly at the brain level to regulate food intake and energy homeostasis in rats [11, 12]. Furthermore, Barb et al. [13] showed that the addition of oleic acid to porcine pituitary cell culture resulted in significantly reduced GnRH-induced LH release compared to those cells cultured without added fat (Control). We hypothesized that the increased interval from calving to ovulation in dairy cows fed a diet supplemented with canola occurred through reduced pituitary responsiveness to hypothalamic GnRH. Therefore, the objective of this study was to determine the effects of prepartum diets supplemented with rolled canola or sunflower seed on LH pulsatility and GnRH-induced LH release during early postpartum period in lactating dairy cows.

## Methods

### Study design and experimental diets

This study was conducted at the Dairy Research Unit of the University of Alberta, Edmonton, Canada, from September to December 2012. All animal experimental procedures were approved by the University of Alberta’s Animal Care and Use Committee for Livestock (protocol # 179/03/13, dated 16 April 2012). Animals were cared for in accordance with the Canadian Council of Animal Care Guidelines.

Thirty-one non-lactating pregnant Holstein cows, parity 1 to 5 (8 primiparous, 23 multiparous), were used in the study. Approximately 35d before the expected calving date (wk-5), cows were blocked by body condition score (BCS), parity and expected calving date, and assigned to 1 of 3 dietary treatments [Canola (high in oleic acid), sunflower (high in linoleic acid), or control (no oilseed)]. Diets were offered ad libitum as a total mixed ration containing forage (alfalfa hay and barley silage) and concentrates (Table 1). Cows were fed a diet supplemented with 8 % rolled oilseeds on a dry matter basis. Oilseeds were rolled as described previously [14] before incorporation in the diet. Upon calving, cows were placed on a common ration containing alfalfa hay, barely silage and concentrate balanced for a lactating dairy cow of 690 kg body weight (BW), producing 45 kg milk per day, according to NRC [15] guidelines.

**Table 1:** Ingredient and nutrient composition of prepartum TMR diets and fatty acid content of canola and sunflower seed

	Prepartum diets		
	Control	Canola	Sunflower
Ingredient composition (% DM)			
Barley silage	60.0	60.0	60.0
Alfalfa hay	10.0	10.0	10.0
Ground barley	10.0	10.0	10.0
Soybean hulls	10.0	6.2	3.4
Canola meal	5.0	0.8	3.6
Canola seed^A^	0.0	8.0	0.0
Sunflower seed^B^	0.0	0.0	8.0
Vitamin / mineral supplements	5.0	5.0	5.0
Nutrient Composition (% DM)			
Crude protein	14.2	13.8	14.4
NDF	42.0	41.3	40.1
Crude fat	2.7	7.4	6.2
Net energy for lactation (Mcal/kg)	1.4	1.6	1.5

^A^Total fat: 45.2 %; Fatty acid content (% of total fat): 61.2 % oleic, 18.8 % linoleic, 9.6 % linolenic, 10.4 % other

^B^Total fat, 43.3 %; Fatty acid content (% of total fat): 12.5 % oleic, 73.1 % linoleic, 0.7 % linolenic, 13.7 % other

Cows were housed individually in tie-stalls during pre and postpartum periods, fed once daily at 0800 h and had unrestricted access to water. Postpartum, cows were allowed 1 to 2 h of exercise daily on week days, and milked twice daily in their stalls between 0400 and 0600 h and between 1530 and 1730 h. Milk production was automatically recorded at each milking and milk samples were collected only during second week postpartum from Tuesday P.M. to Friday A.M. to evaluate milk composition for energy balance calculation. Feed intake was recorded daily and weekly feed samples were taken from forages and concentrates to determine feed dry matter and diet composition [16]. Rations were adjusted weekly based on dry matter content.

Body weight (BW) and BCS were determined before dietary treatments began (wk-5), immediately after calving (wk0) and 5 weeks after calving (wk5). Prepartum BW measures were absolute, with no adjustments made for conceptus weight. The same technician assessed and assigned BCS to each cow, using a scale of 1 (emaciated) to 5 (overconditioned) [17]. Energy balance during pre and postpartum periods was calculated as described by Rabelo et al., [18] with a modification that instead of using a fixed calf weight of 40 kg, actual calf weights were used for calculating energy requirement of pregnancy.

Fatty acid content of oilseeds was determined at the Agricultural Experiment Station Chemical Laboratories (University of Missouri-Columbia, Columbia, MO) and presented in Table 1.

### Blood sampling

Blood samples were collected 2 weeks after initiation of prepartum diets (wk-3), at calving (wk0), first (wk1) and second week postpartum (wk2) from all cows. Samples were collected at 8 h intervals over a 16 h period (2200, 0600 and 1400 h) in heparinized tubes (Vacutainer®, Beckton Dickinson, Franklin Lakes, NJ, USA), centrifuged and plasma harvested. Plasma samples from 2200 and 0600 h were kept at 4 °C until last sample collection at 1400. A pooled sample was prepared by mixing equal quantities of plasma from the 3 consecutive collections and stored at −20 °C until analyzed for non-esterified fatty acids (NEFA), β-hydroxy butyric acid (BHBA) and glucose.

**Fig. 1:**
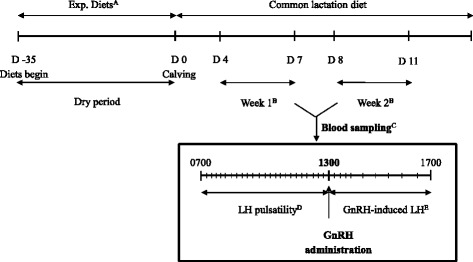
Experimental design and blood sample collection timelines. A. During dry period cows were fed diets supplemented with rolled canola (8 %), or sunflower (8 %) or control (no oilseed). B. During first and second week after calving, blood sample collection was performed to evaluate metabolites, LH pulsatility and GnRH-induced LH release. Cows sampled during first week (n = 5 cows per treatment) for LH pulsatility and GnRH-induced LH were not used in second week sampling (n = 5 or 6 cows per treatment). Blood samples for metabolites were taken from all cows during both weeks. C. Blood sampling design to evaluate LH pulsatility and GnRH-induced LH during first and second week postpartum. D. Blood was sampled every 15 min for 6 h to assess LH pulsatility. E. Blood samples were taken for 4 h, at 15 min intervals during first hour and then at 30 min intervals to evaluate GnRH-induced LH

The blood sample collection schedule for LH determination is summarized in Fig. 1. Cows were assigned to two groups for blood sampling after parturition: wk1 (6 ± 1.0 d, n = 5 per treatment) or wk2 (9 ± 1.2 d, n = 5 or 6 per treatment). Cows sampled during wk1 for LH pulsatility and GnRH-induced LH were not used in wk2 sampling. Blood samples were collected (via indwelling jugular catheter) for 6 h, from 0700 to 1300 at 15 min intervals to assess LH pulsatility. Thereafter, GnRH (100 μg gonadorelin acetate, Fertiline®; Vétoquinol N.-A. Inc., Lavaltrie, QC, Canada) was administrated i.m. (1300 h) and blood was sampled for an additional 4 h, from 1300 to 1700 to determine GnRH-induced LH release. Blood samples were collected at 15 min intervals during the first hour (1300 to 1400) and then at 30 min intervals for the remaining 3 h. Samples were collected in sodium heparinized tubes (Vacutainer®, Beckton Dickinson), and immediately placed on ice until centrifugation (3000 × g for 20 min at 4 °C) within 3 h of collection. Plasma was harvested and stored at −20 °C until assayed.

### Plasma LH and metabolites determination

Plasma LH concentrations were measured by radioimmunoassay using an anti-bovine LH monoclonal antibody (518B7; Quidel Corporation, San Diego, CA, USA). Plasma NEFA (NEFA-C kit, Wako Chemicals USA Inc., Richmond, VA), BHBA (Roche Diagnostics, Indianapolis, IN, USA) and glucose (P7119, Sigma-Aldrich, St. Louis, MO) were determined using commercially available kits. The intra-assay and inter-assay coefficients of variation for NEFA, BHBA and glucose were 1.49 and 3.32, 2.51 and 6.71, and 1.38 and 2.42 %, respectively.

### Statistical analyses

Body weight, BCS, plasma NEFA, BHBA and glucose concentrations as well as energy balance were analyzed using the MIXED procedure of SAS (version 9.3, 2011; SAS Institute Inc., Cary, NC) for repeated measures with unstructured (UN) covariate structure. The final statistical model included dietary treatment, sampling time and parity as the main effects and dietary treatment by sampling time as interaction. Moreover, dry matter intake was analyzed with the same statistical model but First-order Ante Dependence [ANTE (1)] was used as covariance structure. All data are reported as mean ± SE; probabilities < 0.05 were considered significant, whereas those > 0.05 but < 0.10 were considered trends.

The PC-Pulsar program [19] was used to assess LH mean, maximum and minimum concentration (ng/mL), and pulse frequency/6 h and amplitude (ng/mL). Treatment differences were analyzed (n = 5 or 6 per treatment per week) using the MIXED procedure. The statistical model contained treatment, week and parity as the main effects and treatment by week as interaction.

Plasma concentrations of GnRH-induced LH were analyzed (n = 5 or 6 per treatment per week) with the MIXED procedure for repeated measures with Heterogeneous Autoregressive [(ARH(1)] as the covariate structure. Statistical model comprised treatment, week, sampling time and parity as the main effects and treatment by sampling time by week as interaction. The average of LH concentration in last 2 collected samples before GnRH administration was used as covariate in the model. Moreover, the peak concentration of LH and peak-time were chosen from each animal’s raw dataset and then treatment difference was evaluated in created dataset with MIXED procedure. Model consisted of treatment, week and parity as the main effects and the treatment by week as interaction. Area under the curve (AUC) and incline rate were defined as below:

AUC = 1/2∑X_i-1_ (Y_i-1_ + Y_i_), where Xi and Yi are sampling time and LH concentration (ng/mL), respectively [20]. Incline rate = (Peak concentration– Basal concentration)/Time (min).

## Results

### Body condition score, body weight, dry matter intake and energy balance

**Fig. 2:**
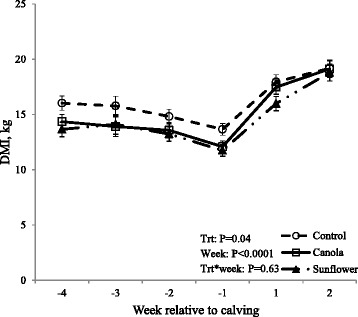
Effects of prepartum dietary treatments on dry matter intake (DMI). Thirty-one cows (n = 10 or 11 per treatment) were fed experimental diets during the last five weeks of gestation. Cows fed control diet consumed more feed than those in sunflower and also tended to consume more feed than those in canola during entire experimental period

**Fig. 3:**
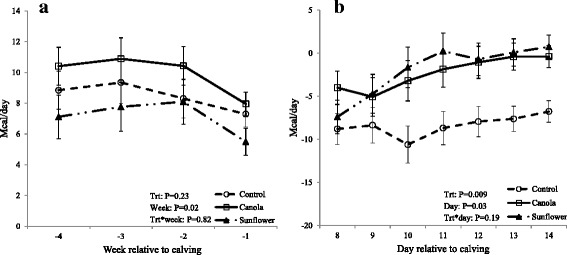
Effect of prepartum diets on energy balance (Mcal/day) during last 4 weeks of gestation and second week postpartum. Thirty-one cows (n = 10 or 11 per treatment) were fed experimental diets during the last five weeks of gestation. Prepartum diets did not influence the energy balance during last 4 weeks of gestation (**a**). The average of energy balance during last 4 weeks of gestation was 9.92 ± 1.05, 8.44 ± 1.08 and 7.11 ± 1.22 Mcal/day in canola, control and sunflower, respectively. Regardless of treatment effect, energy balance during last week of gestation was significantly reduced compared to wk–4, –3, and –2. Prepartum diets significantly affected postpartum energy balance during the second week (**b**). Cows fed control diet had a more pronounced negative energy balance than those fed diets supplemented with canola or sunflower. Energy balance was significantly higher on Day 14 than on Days 11, 10, 9 and 8

There was no difference in BCS or BW among treatments at initiation of prepartum diets (BCS wk-5 and BW wk-5). Prepartum dietary treatments did not affect BCS (P = 0.17) and BW (P = 0.85) at calving (BCS wk0 and BW wk0) or 5 weeks after calving (BCS wk5 and BW wk5). Likewise, BCS between wk-5 and wk0 (P = 0.82), as well as between wk0 and wk5 (P = 0.36) did not differ (Table 2). Dry matter intake (DMI) significantly differed (P = 0.04) among dietary treatments (Fig. 2) during the entire experimental period; cows fed control diet consumed more (16.23 ± 0.49 kg) than those fed sunflower seed (14.58 ± 0.47 kg; P = 0.01) and tended to consume more than those fed canola seed (15.08 ± 0.64 kg; P = 0.08). Further analysis of pre and postpartum DMI as separate dataset indicated that during the prepartum period, cows fed the control ration consumed more (15.30 ± 0.63 kg) than those fed sunflower (13.31 ± 0.57 kg; P = 0.01) or canola (13.54 ± 0.55 kg; P = 0.03). However, postpartum DMI did not differ among dietary treatments (mean, 17.50 kg; P = 0.37). Moreover, prepartum diets did not affect (P = 0.23) energy balance during the last 4 weeks of gestation (Fig. 3a), whereas postpartum energy balance (Fig. 3b) was affected (P = 0.009) by prepartum dietary treatments. Cows on a control diet (−8.39 ± 1.52 Mcal/day) were in a deeper state of negative energy balance than those fed diets supplemented with canola (−2.28 ± 1.60 Mcal/day; P = 0.03) or sunflower (−1.91 ± 1.63 Mcal/day; P = 0.01) during wk2 postpartum.

**Table 2:** Effects of prepartum diets on body condition score (BCS) and body weight (BW)

	Prepartum diets			*P*	
	Control	Canola	Sunflower	Trt^A^	Trt*wk^B^
	(n = 11)	(n = 10)	(n = 10)		
Body condition score				0.17	0.80
BCS (wk-5)	3.28 ± 0.06	3.28 ± 0.06	3.39 ± 0.06		
BCS (wk0)	3.44 ± 0.06	3.49 ± 0.06	3.53 ± 0.05		
BCS (wk5)	2.96 ± 0.08	3.00 ± 0.07	3.16 ± 0.07		
BCS difference wk-5 & 0	0.15 ± 0.09	0.20 ± 0.09	0.15 ± 0.08	0.82	-
BCS difference wk0 & 5	−0.49 ± 0.09	−0.48 ± 0.09	−0.33 ± 0.08	0.36	-
Body weight				0.85	0.61
BW (wk-5)	642.5 ± 24.7	621.1 ± 22.9	634.4 ± 23.5		
BW (wk0)	642.5 ± 23.0	613.0 ± 22.8	631.71 ± 21.9		
BW (wk5)	557.8 ± 23.4	562.0 ± 21.1	564.6 ± 21.7		
BW difference wk-5 & 0	−3.2 ± 17.8	−3.1 ± 18.8	−2.9 ± 17.2	0.99	-
BW difference wk0 & 5	−75.6 ± 18.3	−44.1 ± 19.3	−62.8 ± 17.6	0.45	-

^A^Trt: Dietary treatment

^B^wk: Postpartum week

### Plasma metabolites

Concentrations of plasma metabolites (NEFA, BHBA and glucose) are summarized in Table 3. Mean NEFA concentration tended (P = 0.07) to be higher in canola than control at wk-3 but it was not different from sunflower. During the postpartum period, cows fed control diet had greater NEFA concentration at second week compared with those fed sunflower (P = 0.03) and tended (P = 0.10) to be higher than those fed canola. Prepartum dietary treatments did not affect overall mean concentration of BHBA and glucose.

**Table 3:** Effects of prepartum diets on plasma metabolites concentration

	Prepartum diets			*P*	
	Control	Canola	Sunflower	Trt^A^	Trt*wk^B^
	(n = 11)	(n = 10)	(n = 10)		
NEFA(mEq/dL)				0.40	0.05
wk-3	81 ± 5^¥^	95 ± 5^¥^	85 ± 5		
wk0	507 ± 54	397 ± 61	441 ± 54		
wk1	392 ± 48	439 ± 50	453 ± 50		
wk2	584 ± 64^§a^	428 ± 68^§ab^	381 ± 68^b^		
BHBA (mg/dL)				0.28	0.42
wk-3	11.3 ± 0.5	9.9 ± 0.5	9.7 ± 0.5		
wk0	10.1 ± 0.8	10.6 ± 0.8	10.3 ± 0.8		
wk1	12.0 ± 1.0	12.9 ± 1.0	11.2 ± 1.0		
wk2	14.2 ± 1.1	11.9 ± 1.1	11.7 ± 1.1		
Glucose (mg/dL)				0.50	0.78
wk-3	56.3 ± 1.4	58.1 ± 1.3	58.7 ± 1.3		
wk0	77.9 ± 5.3	75.9 ± 5.5	80.6 ± 5.5		
wk1	49.8 ± 1.5	48.7 ± 1.5	50.9 ± 1.5		
wk2	46.0 ± 1.5	47.7 ± 1.5	50.4 ± 1.5		

^a,b^: P = 0.03;^¥^ Control tended (P = 0.07) to have lower NEFA concentration than canola; ^§^Control tended (P = 0.10) to have greater NEFA concentration than canola

^A^Trt: Dietary treatment

^B^wk: Postpartum week

wk-3: 2 weeks after the initiation of prepartum diets; wk0: calving; wk1: first week postpartum; wk2: second week postpartum

### LH pulsatility

Dietary treatments did not affect mean, maximum and minimum concentrations of LH, LH pulse frequency or amplitude during wk1 and wk2 postpartum (Table 4). Likewise, LH pulsatility was not affected by postpartum week.

**Table 4:** Characteristics of LH pulsatility^A^ among prepartum dietary treatments

	Week 1^B^			Week 2^B^			*P*	
	Control	Canola	Sunflower	Control	Canola	Sunflower	Trt^C^	Trt*wk^D^
Cows (n)	5	5	5	6	5	5		
LH Mean	0.25 ± 0.14	0.25 ± 0.15	0.28 ± 0.13	0.24 ± 0.14	0.54 ± 0.14	0.28 ± 0.14	0.53	0.51
LH Min	0.11 ± 0.06	0.10 ± 0.06	0.09 ± 0.05	0.11 ± 0.07	0.16 ± 0.07	0.10 ± 0.07	0.83	0.92
LH Max	0.62 ± 0.39	0.63 ± 0.42	1.27 ± 0.37	0.61 ± 0.39	1.49 ± 0.39	0.69 ± 0.39	0.74	0.87
Frequency	3.91 ± 0.65	4.15 ± 0.69	4.75 ± 0.60	3.45 ± 0.63	4.95 ± 0.63	4.25 ± 0.63	0.30	0.55
Amplitude^E^	0.38 ± 0.10	0.39 ± 0.10	0.44 ± 0.09	0.33 ± 0.09	0.52 ± 0.09	0.36 ± 0.09	0.61	0.54

^A^Blood samples were collected every 15 min for 6 h

^B^Week 1: First week postpartum; Week 2: Second week postpartum; ^C^Trt: Dietary treatment; ^D^wk: Postpartum week; ^E^The amplitude of LH pulses is defined as the height from the preceding nadir to maximum height

### GnRH-induced LH release

**Fig. 4:**
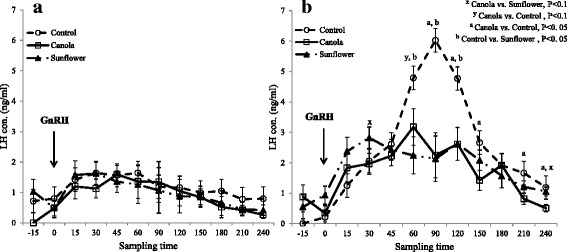
Effect of prepartum diets on GnRH-induced LH release during first and second week postpartum. Cows were fed experimental diets during the last five weeks of gestation and assigned for GnRH-induced LH measurement in postpartum wk1 (n = 5 per treatment) or wk2 (n = 5 or 6 per treatment). All cows responded to the GnRH treatment and had higher (P < 0.05) LH concentrations from 15 to 60 min post-treatment. However, prepartum dietary treatments did not affect GnRH-induced LH release pattern during wk1 postpartum (**a**). Prepartum diets significantly influenced GnRH-induced LH concentrations during wk2 postpartum (**b**). All cows responded to GnRH treatment, and LH concentrations remained elevated (P < 0.05) for up to 240 min post-treatment. Cows fed control (no oilseed) diet prepartum released more LH at 60, 90 and 120 min after GnRH administration than those fed sunflower or canola during wk2. The treatment by sampling time by week interaction was significant (P < 0.03)

Irrespective of prepartum dietary treatment, the mean LH (ng/ml) concentration after GnRH administration was increased (P < 0.0001) from wk1 (0.98 ± 0.09) to wk2 (1.97 ± 0.09) postpartum. Both at wk1 and wk2 postpartum, cows responded to exogenous GnRH administration by increasing LH release by 15 min post treatment (P < 0.01; Fig. 4a and b). In wk1, GnRH-induced LH concentrations (ng/mL) were higher only from 15 to 60 min post-GnRH (mean, 1.44 ± 0.20), relative to LH at time zero (0.58 ± 0.15). However, in wk2, GnRH-induced mean LH concentrations remained higher than at time zero (0.49 ± 0.15) from 15 min to the end of sampling at 240 min (2.27 ± 0.10). During wk1 postpartum, GnRH-induced LH release did not differ among dietary treatments, but during wk2 postpartum the mean LH released in cows fed a prepartum diet supplemented with canola (1.66 ± 0.23 ng/mL) was lower (P = 0.02) than that in those fed a control diet (2.42 ± 0.21 ng/mL). Cows fed a diet supplemented with sunflower tended (P = 0.09) to release less LH (1.83 ± 0.18 ng/mL) than those fed a control diet (Table 5). The interaction of dietary treatment by sampling time by wk postpartum indicated that there was no difference among dietary treatment by sampling time during wk1 (Fig. 4a). However, cows fed a control diet released more LH at 60, 90 and 120 min after GnRH administration than those fed either sunflower or canola during wk2 (Fig. 4b). Prepartum dietary treatments also affected AUC (ng/mL per 4 h) during wk2 postpartum, which was greater (P = 0.01) in control cows compared to those fed sunflower seed and tended (P = 0.08) to be greater than those fed canola. In addition, mean AUC of LH was higher (P = 0.007) in wk2 postpartum (143.33 ± 14.85) than in wk1 (58.23 ± 17.85). However, dietary treatments did not affect LH peak concentrations, LH peak-time, and incline rate during wk1 and wk2 postpartum (Table 5).

**Table 5:** Effects of prepartum diet on GnRH-induced LH release during early postpartum

	Week 1^A^			Week 2^A^			*P*	
	Control	Canola	Sunflower	Control	Canola	Sunflower	Trt^B^	Trt*wk^C^
Cows (n)	5	5	5	6	5	5		
LH Mean (ng/mL)	1.14 ± 0.14	0.84 ± 0.23	0.96 ± 0.21	2.42 ± 0.21^¥a^	1.66 ± 0.23^b^	1.83 ± 0.18^¥ab^	0.03	0.05
LH- Peak (ng/mL) ^D^	2.05 ± 0.75	3.21 ± 0.80	2.19 ± 0.70	5.14 ± 0.80	4.07 ± 0.73	3.72 ± 0.73	0.37	0.11
LH-Peak-time (min) ^E^	32.50 ± 13.26	48.00 ± 13.78	22.50 ± 10.27	81.37 ± 21.53	101.63 ± 21.53	63.37 ± 21.53	0.48	0.74
AUC (ng/mL per 4 h) ^F^	41.52 ± 27.39	84.65 ± 29.26	48.51 ± 25.38	197.26 ± 26.74^§c^	125.73 ± 26.74^§cd^	106.99 ± 26.74^d^	0.26	0.06
Incline rate (ng/mL per min) ^H^	0.09 ± 0.02	0.09 ± 0.02	0.08 ± 0.02	0.06 ± 0.02	0.07 ± 0.02	0.07 ± 0.02	0.98	0.97

^a,b^: P = 0.02; ^c,d^: P = 0.01

^¥^Control tended (P = 0.09) to have greater LH mean concentration than sunflower at wk2. ^§^Control tended (P = 0.08) to have greaterAUC than canola at wk2. ^A^Week1: First week after calving (n = 5 cows per treatment); Week2: Second week after calving (n = 5 or 6 cows per treatment); ^B^Trt: Dietary treatment; ^C^wk: Postpartum week; ^D^Peak: The highest concentration of LH after GnRH administration; ^E^Peak-time (min): The interval in minutes from GnRH administration to LH peak; ^F^AUC: Area under curve = 1/2∑X_i-1_ (Y_i-1_ + Y_i_), where Xi and Yi are sampling time and LH concentration (ng/ml), respectively; ^H^Incline rate = (peak concentration– basal concentration)/Time (min)

### Ovarian follicle size

Prepartum diets did not affect the size of the largest ovarian follicle in either of the two postpartum weeks (Table 6).

**Table 6:** The mean diameter of the largest ovarian follicle in postpartum weeks 1 and 2 in each of the three dietary groups

Prepartum diet	Mean ± S.E. of follicle diameter (mm)	
	Week 1^A^	Week 2^A^
Control	9.41 ± 1.34	13.90 ± 1.47
Canola	6.40 ± 1.47	13.80 ± 1.47
Sunflower	7.00 ± 1.47	10.90 ± 1.47

^A^Week1: First week after calving (n = 5 cows per treatment); Week2: Second week after calving (n = 5 or 6 cows per treatment). Follicle size was not influenced by treatment (P = 0.18) or treatment x week interaction (P = 0.45)

## Discussion

In dairy cows, energy balance is one of major factors affecting LH pulse frequency and resumption of ovarian activity during early postpartum [7, 8, 21, 22]. In the current study, lower net energy in the control diet resulted in higher dry matter intake compared to those fed diets supplemented with rolled sunflower or canola seed during the prepartum period. Cows fed a diet supplemented with canola seed tended to have higher NEFA at wk-3 than those fed control possibly due to lower feed intake during prepartum period. Although, prepartum dietary treatments did not affect postpartum DMI, cows fed a control diet prepartum were in a deeper state of negative energy balance and had higher NEFA concentrations during wk2 postpartum than those fed prepartum diets supplemented with sunflower or canola. In previous studies, restricting energy intake increased peak and total GnRH-induced LH release through reduced LH pulsatility [23, 24]. Therefore, a more pronounced negative energy balance in control treatment during wk2 postpartum may have contributed to the increased GnRH-induced LH release in cows of that group compared to those fed oilseeds, albeit LH pulse frequency in the control group was not significantly lower than in cows fed oilseeds.

The possibility that cows on the control diet had larger ovarian follicles and higher estradiol concentrations in wk2, consequently resulting in higher GnRH-induced LH release was considered. However, as evident from Table 6, we found no significant influence of diets on follicle size in either of the two postpartum weeks. Although estradiol concentrations were not measured in this study, it is highly unlikely that estradiol was the contributing factor for the increased release of GnRH-induced LH in the control group.

The mean basal (pre-GnRH treatment) LH concentration during wk1 postpartum (0.26 ng/mL) was lower than what was previously reported (1.5 ng/ml) in one study [25], but agrees with another report in early postpartum dairy cows (0.32 ng/ml; [26]). Neither the first nor second postpartum week influenced LH pusatility in the present study irrespective of dietary treatment. Prepartum diets did not affect LH mean, maximum and minimum concentration, pulse frequency or pulse amplitude, which was likely due to low pituitary reserves of LH [26–28]. To identify endocrine events that may influence the duration of postpartum anestrus, Moss et al. [28] slaughtered mature beef cows at 5, 10, 20 or 30 d after calving and another group at 12 to 14 d after their first postpartum estrus. They found that tissue concentrations of pituitary LH were low during early postpartum (at 5, 10 and 20 d) but significantly increased to levels comparable to luteal phase concentrations by 30 d postpartum. However, neither the GnRH receptor populations nor the affinity of anterior pituitary receptors for GnRH differed among the postpartum groups. In the same study, anterior pituitary cells from 5 d postpartum released significantly less LH in response to GnRH, when cultured in vitro, than those from luteal phase cows, but GnRH-induced LH release from pituitary cells in vitro in cows that were 10, 20 or 30 d postpartum did not differ from luteal phase cows. Thus, the replenishment of pituitary stores of LH may be one of the initial limitations to the reestablishment of reproductive competence after calving [28]. Other studies have shown that pituitary LH content in dairy cattle increased during the postpartum period, but was not fully restored until approximately 19 d postpartum [26, 27].

More recently, Garrel et al. [29] infused a triglyceride emulsion (containing 61 % linoleic and linolenic acids) with heparin, through carotid catheter to rat brain for 24 h. Heparin was used to stimulate lipoprotein lipase activity and thus to release fatty acids from triglyceride. The infusion of the fatty acid emulsion did not affect serum LH concentrations in the above study. However, adding oleic and linoleic acid to rat [29] and pig [13] pituitary cell culture increased LH concentrations by enhancing LHβ gene expression. The observed discrepancies between in vivo and vitro studies in LH secretion could be due to differences in concentrations and/or the mechanics of fatty acid delivery to gonadotropes, or due to species differences.

In the current study, independent of treatment effect, GnRH-induced LH release significantly increased from wk1 to wk2 postpartum, in agreement with previous studies [25, 26], but it was not affected by prepartum dietary treatments during wk1 postpartum. Moss et al. [28] indicated that the numbers of anterior pituitary receptors for GnRH at day 5 postpartum were the same as at 10, 20 or 30 d after calving. Therefore, the lower pituitary responsiveness to exogenous GnRH during wk1 postpartum (6 ± 1.0 d) was likely due to low pituitary LH content. Our finding that cows in wk2 postpartum released greater quantities of LH, which remained elevated for a longer duration, and had a higher AUC, support this notion. Furthermore, feeding a prepartum diet supplemented with canola seed (high oleic) resulted in lower LH release in response to exogenous GnRH in wk2 compared with those fed control diet, and cows fed a diet supplemented with sunflower seed (high linoleic) tended to release less GnRH-induced LH than those fed a control diet. Binding of GnRH to its receptor, a member of the G protein-coupled receptor family, initiates a wide array of signaling events among which one of them is GnRH mobilization of intracellular Ca_2_^+^. Intracellular Ca_2_^+^ mobilization regulates acute gonadotropin release. Garrel et al. [29] found that the addition of linoleic or oleic acid decreased GnRH signaling as evidenced by a significant reduction in GnRH-induced calcium mobilization in pituitary gonadotropes. It has also been reported [30] that GnRH-induced increase in intracellular Ca_2_^+^ is partly due to stimulation of voltage-gated calcium channels that can be inhibited by polyunsaturated fatty acids.

Cows fed canola and sunflower seed during the late gestation period in the present study, consumed 330 and 380 g of oleic and linoleic acids, respectively, and 485 and 450 g of total long chain fatty acids (oleic, linoleic and linolenic acids combined) daily. Long chain fatty acids consumed daily at these levels for up to 35 d preceding parturition could have had carryover effects interfering with GnRH-induced calcium mobilization in pituitary gonadotropes, thereby affecting LH release, although this remains a speculation at this time.

## Conclusions

Current results show that prepartum diets did not affect pulsatile and GnRH-induced LH release during wk1 postpartum. Although prepartum diets also did not affect LH pulsatility during wk2 postpartum, cows that consumed either canola or sunflower seed prepartum had lower responsiveness to GnRH treatment, releasing less LH than cows fed a control diet. Greater negative energy balance and numerically lower LH pulse frequency in control treatment postpartum may have increased the releasable pool of LH to exogenous GnRH. Previous in vitro results [13, 29] and our present findings suggest that dietary long chain fatty acids (particularly oleic and linoleic) interrupt GnRH-induced LH release. While present findings lend support to our hypothesis that a longer interval from calving to ovulation in dairy cows fed canola seed [5] occurred through suppression of pituitary responsiveness to GnRH, the lack of differences in GnRH-induced LH release between canola (high oleic) and sunflower (high linoleic) diets, still leaves some questions unanswered, warranting further investigations.

## Abbreviations

AUC: Area under curve
BCS: Body condition score
BHBA: β-hydroxy butyric acid
BW: Body weight
DMI: Dry matter intake
GnRH: Gonadotropin releasing hormone
LH: Luteinizing hormone
NEFA: Non-esterified fatty acids
